# The Effect of Delayed Oncology Surgery on Survival Outcomes for Patients With Gastric Cancer During the COVID-19 Pandemic: Evidence-Based Strategies

**DOI:** 10.3389/fonc.2022.780949

**Published:** 2022-05-19

**Authors:** Jichun Ma, Chenglou Zhu, Weidong Li, Zhisheng Qiu, Jian Yang, Long Ge, Mingxu Da

**Affiliations:** ^1^ The First Clinical Medical College of Lanzhou University, Lanzhou University, Lanzhou, China; ^2^ Clinical Medicine College, Ningxia Medical University, Yinchuan, China; ^3^ Department of Oncology Surgery, Gansu Provincial Hospital, Lanzhou, China; ^4^ Evidence-Based Social Science Research Center, School of Public Health, Lanzhou University, Lanzhou, China

**Keywords:** gastric cancer, surgery, COVID-19, overall survival, delay

## Abstract

**Objective:**

To evaluate the impact of delay in gastrectomy on gastric cancer patients’ survival outcomes during the COVID-19 pandemic.

**Methods:**

Databases including PubMed, MEDLINE (using the Ovid platform), Embase, the Cochrane Library, COVID-19 Open Research Dataset Challenge, COVID-19 Research Database (WHO), ClinicalTrials.gov, and WHO International Clinical Trials Registry Platform were searched for studies of any design and in any setting that included patients with gastric cancer from their inception to July 31, 2021. Hazard ratio (HR) and 95% confidence intervals (CI) of research endpoints in each study were calculated. Statistical analyses were performed with Stata 12.0.

**Results:**

A total of 8 studies involving 4,052 gastric cancer patients were eligible and included in the present meta-analysis. The result of the meta-analysis was shown that delaying surgery for less than 8 weeks may not decrease OS (HR = 0.91, 95% CI: 0.80~1.04, p = 0.167) and DFS (HR = 0.96, 95% CI: 0.62~1.50, p = 0.872) in gastric cancer. Our meta-analysis also illustrated that delay in surgery for more than 4 weeks (HR = 0.85, 95% CI: 0.56~1.27, p = 0.421), 6 weeks (HR = 0.88, 95% CI: 0.61~1.27, p = 0.490), and 8 weeks (HR = 0.93, 95% CI: 0.80~1.07, p = 0.314) was also not associated with a decreased OS.

**Conclusion:**

A delay in surgery of less than 8 weeks is not associated with worse overall survival for patients with gastric cancer.

## Introduction

A Coronavirus Disease 2019 (COVID-19) pandemic was declared by WHO on March 11, 2020, which placed an unprecedented threat for thousands of cancer patients by disrupting their timely treatment schedules (1). Hospitals around the world delayed or canceled the elective surgeries to limit the spread of COVID-19 and increase the capacity of systems (2). Patients who are going to have surgery are at a higher risk of COVID-19 exposure in hospitals, due to the immunosuppressive responses and pro-inflammatory cytokines (3). During this time, nearly 38% of oncology operations were estimated to have been delayed and canceled (4). For many malignancies, surgery is a foundation of most curative therapies (5). Delaying the surgery of cancer patients may lose a window for resection, which would lead to a poorer survival benefit for these patients and the need for additional neoadjuvant or adjuvant therapy. Thus, surgeons must critically identify which approaches including surgery and other therapeutic options to delay the cancer surgery are better for patients with cancer.

Although the Society of Surgical Oncology (SSO) and American College of Surgeons (ACS) have published guidelines for surgical procedures of non-emergent surgery, the effect of time to surgery for many malignancies still has not been well characterized and the “acceptable” waiting time prior to oncology surgery to a worsened clinical outcome is unclear (6, 7). The aim of the present study is to provide evidence-based information to support the decision-making for the surgical management of gastric cancer (GC) and investigate the impact of delaying the surgery on the survival benefit for GC patients.

## Methods

### Search Strategy

We searched the databases including PubMed, MEDLINE (using the Ovid platform), Embase, the Cochrane Library, COVID-19 Open Research Dataset Challenge, COVID-19 Research Database (WHO), ClinicalTrials.gov, and WHO International Clinical Trials Registry Platform for studies of any design and in any setting that included patients with gastric cancer from their inception to July 31, 2021. The search strategy included the following specific terms: “COVID-19” OR “nCoV” OR “sarscov 2” OR “2019nCoV” OR “novel CoV” OR “SARS-CoV-2”, “gastric cancer” OR “gastric carcinoma” OR “stomach cancer” OR “stomach neoplasm”, “surgery” OR “surgical procedures” OR “operative surgical procedures” OR “general surgery”, We did not limit our search by ethnicity and language.

### Study Inclusion and Data Collection

Studies were included if the researchers evaluated the impact of delaying the surgery on the survival benefit for GC patients. Studies were excluded if they were abstract, animal experiment, or patients under 18 years of age.

The data were extracted from included studies by two researchers independently, which was the following: (1) the characteristics of included studies, including the first author, the year of publication, country, gender, sample size, and mean age of patients and (2) clinical outcomes, including overall survival (OS) and disease-free survival (DFS).

### Assessment of Study Quality

We used the Newcastle–Ottawa scale (NOS) (8) to assess the quality of included studies: (1) the selection of cohorts (0–4 points); (2) comparability of cohorts (0–2 points); and (3) the exposure or outcome of the participant (0–3 points). Finally, the total score of each study represented the overall result of quality assessment. Studies with 7 to 9 points were regarded as “high quality”.

### Data Analysis

The present meta-analysis was conducted by the STATA version 12.0. The heterogeneity between included studies was evaluated by the I^2^-based Q-test: the fixed-effect model was used to pool the hazard ratio (HR) and its 95% confidence interval (CI) if the *p* value was higher than 0.1 or I^2^ ≤ 50%. Otherwise, the random-effect model was adopted. Subgroup analyses were performed by country, sample size, tumor differentiation, surgical type, surgical approach, and time delayed to surgery. Potential publication bias was measured by funnel plots. All p values were two-sided, and statistical significance was accepted as p ≤ 0.05.

## Results

### Study Selection

**Figure 1:**
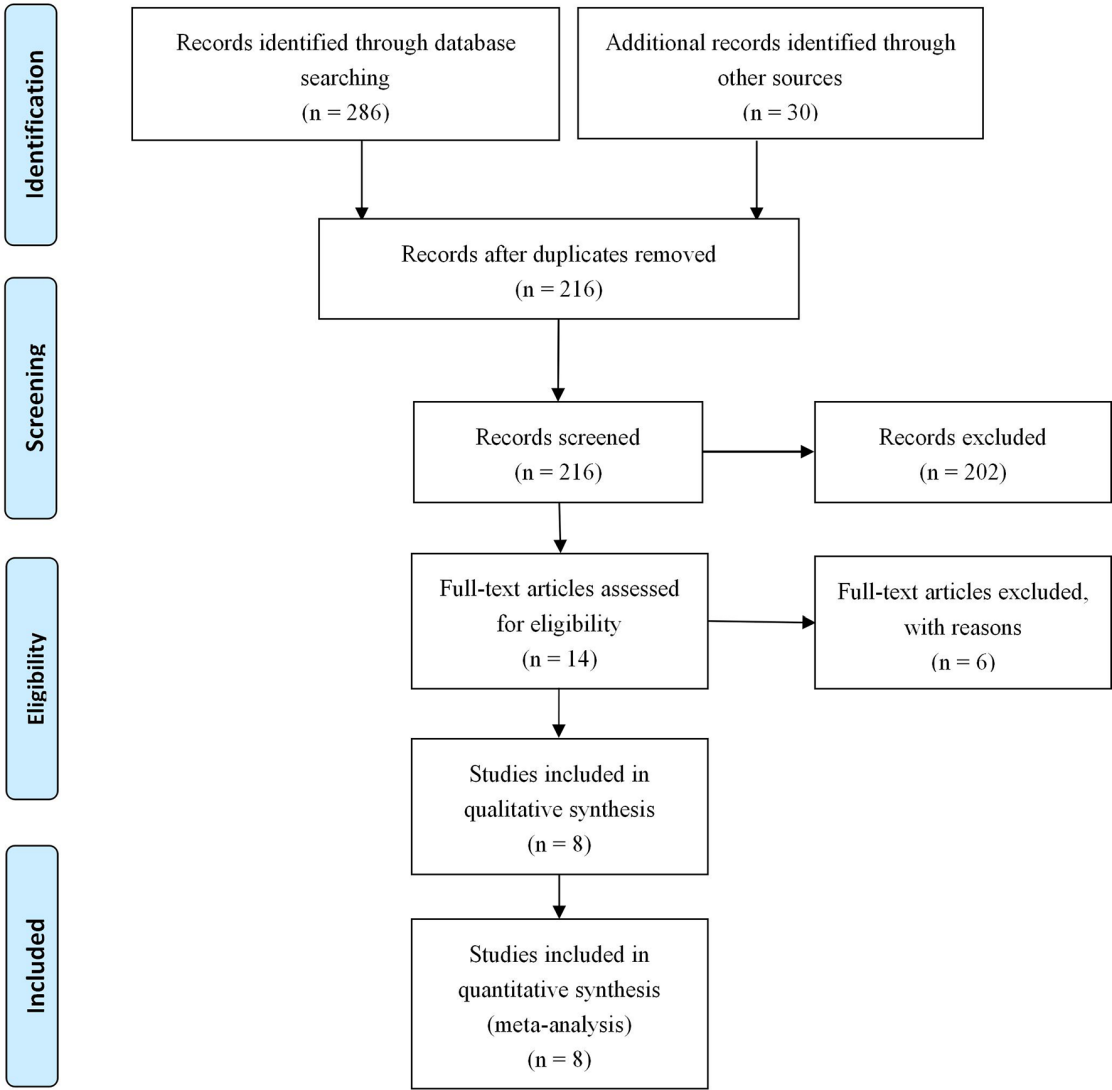
PRISMA flow diagram of the meta-analysis.

A total of 316 abstracts were identified from the search strategy. After screening of these abstracts, 8 studies (9–16) involving 4,052 gastric cancer patients were eligible and included in the present meta-analysis. Detailed information about the flowchart of the study selection process is reported in **Figure 1**.

### The Baseline of Included Studies

All of the 8 included studies are retrospective studies. Years of publication of included studies ranged from 2014 to 2021, and sample sizes ranged from 154 to 1,439 patients. Patients’ characteristics and baseline are presented in **Table 1**. Among the studies included in this meta-analysis, three of them are from Korea (3, 37.5%), two of them are from China (2, 25%) and Japan (2, 25%), and one of them is from Netherlands (1, 12.5%). The mean score on the NOS was 8.4 for all of the included studies, which illustrated a high methodological quality of included studies.

**Table 1:** the characteristics of included studies.

Study	Country	Sample size	Age (years)	Gender (F/M, n)	Tumor differentiation (n, %)			Surgical type (n)			Adjuvant therapy (n)			
					Undifferentiated	Moderate	High differentiation	Distal gastrectomy	Total gastrectomy	Multiorgan surgery	No	Chemo	Radio	Chemoradiation
Brenkman (9)	Netherlands	591	63.30 ± 10.5	212/379	272 (46.02)	63 (10.66)	NR	303 (51.27)	273 (46.19)	5	262	246	2	83
			62.40 ± 9.00											
Kim (10)	Korea	154	62.00 ± 9.00	41/113	51 (33.12)	52 (33.77)	60 (38.96)	127 (82.47)	27 (17.53)	0	154	0	0	0
			59.30 ± 10.30											
Liu (11)	China	176	55.56 ± 10.81	74/102	120 (68.18)	39 (22.16)	17 (9.66)	50 (28.41)	126 (71.59)	NR	NR	176	0	0
			59.79 ± 9.79											
Fujiya (12)	Japan	371	64 (57–71)	123/248	185 (49.86)	NR	NR	276 (74.39)	73 (19.68)	NR	NR	NR	NR	NR
			67 (60–75)											
Furukawa (13)	Japan	593	67 (58–74)	197/396	349 (58.85)	244 (41.15)	NR	305 (51.43)	259 (15.56)	1	NR	NR	NR	NR
			68 (60–74)											
Cha (14)	Korea	302	62.04 ± 9.17	87/215	88 (29.14)	125 (41.39)	85 (28.15)	255 (84.44)	47 (15.56)	NR	NR	NR	NR	NR
			61.14 ± 9.76											
Wang (15)	China	426	61 (57–66)	99/327	198 (46.48)	217 (50.94)	11 (2.58)	191 (44.84)	235 (55.16)	NR	77	394	NR	NR
			60 (53–66)											
Na (16)	Korea	1439	61 (22–88)	372/1067	322 (22.38)	1117 (77.62)	NR	NR	NR	NR	NR	NR	NR	NR
			61 (32–88)											

NR, not reported; Chemo, chemotherapy; Radio, radiotherapy.

### Meta-Analysis of OS

**Figure 2:**
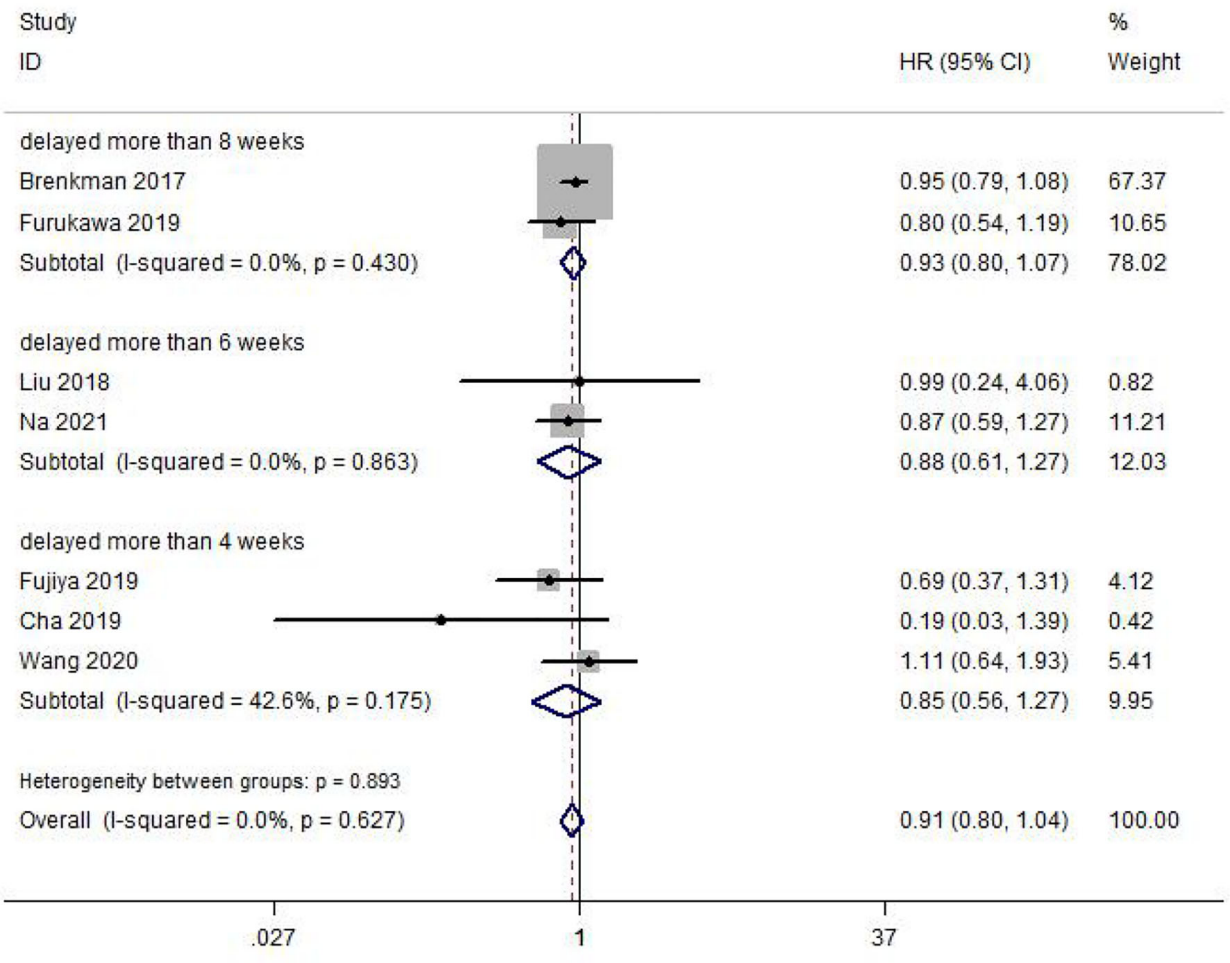
Meta-analysis of the estimated hazard ratio for overall survival for a delay in gastric cancer surgery.

A total of 7 included studies reported the OS impacted by delayed surgery time for GC patients. Heterogeneity between studies was not significant (I^2^ = 0.0%, p = 0.627). Meta-analysis was performed with a fixed-effect model. The result of the meta-analysis was shown that delaying surgery for less than 8 weeks may not decrease OS in gastric cancer (HR = 0.91, 95% CI: 0.80~1.04, p = 0.167) (**Figure 2**). Our meta-analysis also illustrated that delay in surgery for more than 4 weeks (HR = 0.85, 95% CI: 0.56~1.27, p = 0.421), 6 weeks (HR = 0.88, 95% CI: 0.61~1.27, p = 0.490), and 8 weeks (HR = 0.93, 95% CI: 0.80~1.07, p = 0.314) was also not associated with a decreased OS. Subgroup analysis by country, sample size, tumor differentiation, surgical type, and surgical approach was also conducted; all of them showed no significant difference in this meta-analysis (**Table 2**).

**Table 2:** Subgroup analysis of OS.

Items	No. of studies	HR and 95% CI	p value
Country	7	0.91 (0.80,1.04)	0.627
China	2	1.09 (0.65,1.83)	0.883
Japan	2	0.77 (0.55,1.07)	0.695
Korea	2	0.82 (0.57,1.20)	0.142
Netherlands	1	0.95 (0.81,1.11)	0.111
Sample size	7	-0.90 (-0.219,0.038)	0.627
≤500	3	-0.083 (-0.219,0.053)	0.698
>500	4	-0.155 (-0.546,0.236)	0.317
Tumor differentiation	7	-0.90 (-0.219,0.038)	0.627
Undifferentiated ≤ 40%	2	-0.194 (-0.570,0.182)	0.142
Undifferentiated > 40%	5	-0.077 (-0.213,0.060)	0.758
Surgical type	6	-0.084 (-0.221,0.052)	0.507
Distal gastrectomy ≥ 50%	4	-0.097 (-0.239,0.044)	0.283
Total gastrectomy ≥ 50%	2	0.089 (-0.425,0.603)	0.883
Surgical approach	3	-0.079 (-0.230,0.072)	0.119
Open ≥50%	2	-0.070 (-0.221,0.082)	0.336
Laparoscopic ≥ 50%	1	-1.645 (-3.617,0.327)	0.186

OS, overall survival; HR, hazard ratio; 95% CI, 95% confidence interval.

### Meta-Analysis of DFS

**Figure 3:**
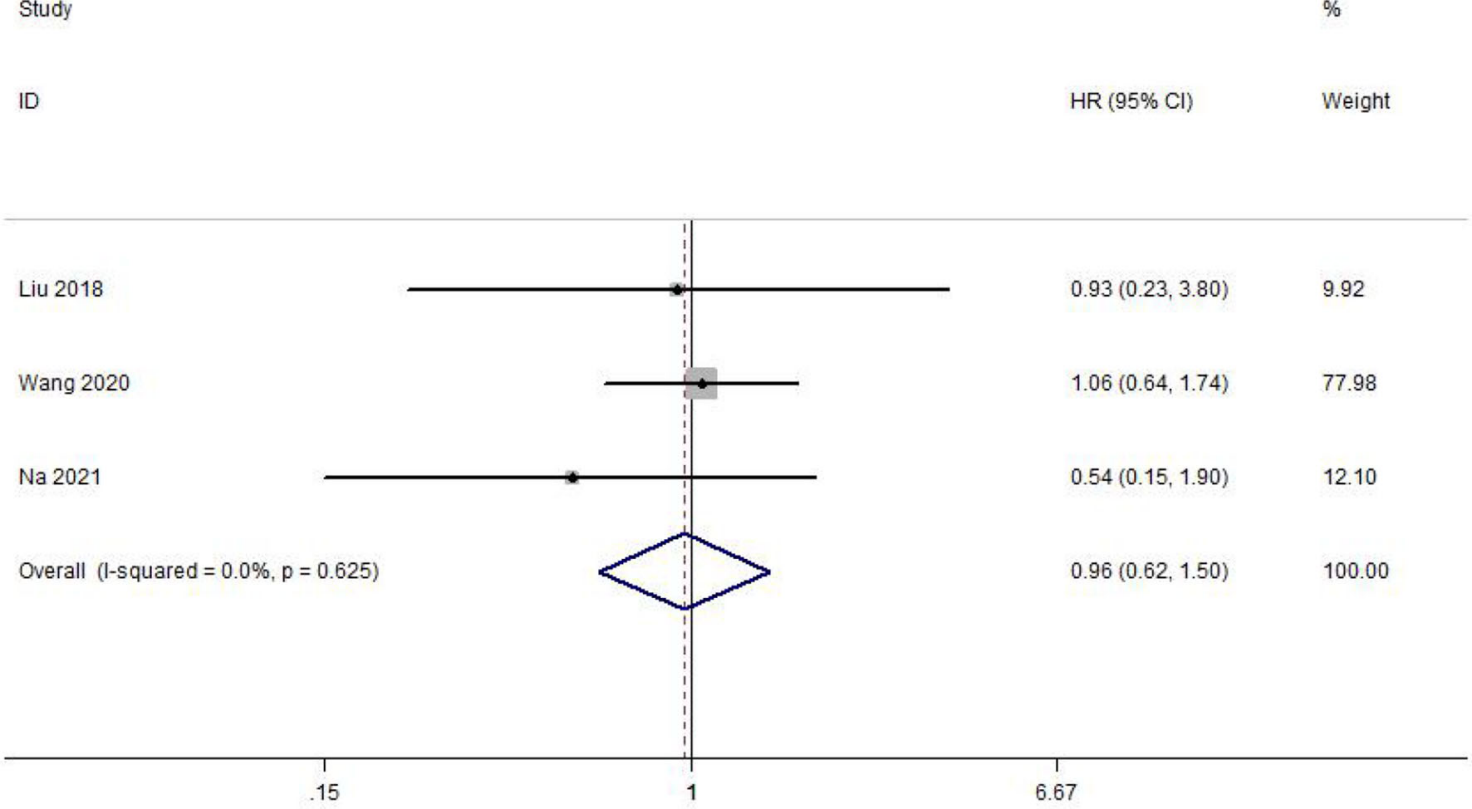
Meta-analysis of the estimated hazard ratio for disease-free survival for a delay in gastric cancer surgery.

DFS was reported in 3 of the included studies. Heterogeneity between studies was not significant (I^2^ = 0.0%, p = 0.625). Meta-analysis was performed with a fixed-effect model. The result of the meta-analysis was shown that delaying surgery for less than 8 weeks may not decrease DFS in gastric cancer (HR = 0.96, 95% CI: 0.62~1.50, p = 0.872) (**Figure 3**).

## Discussion

Due to the immunocompromised status for patients with cancer, they were considered more susceptible to be infected by SARS-CoV-2 (17). Anticancer treatments such as surgery, chemotherapy, radiotherapy, and immunotherapy cannot be conducted on time (18). Given this, oncology patients undergoing anticancer therapy during the COVID-19 pandemic should be treated in balance (19). Currently, there is a lack of evidence-based strategies for what is considered a delay in gastric cancer surgery. Few studies attempted to examine if surgical delays impact the survival for cancer patients. Fligor et al. (20) suggested that delayed oncology resection of colorectal cancer worsens patients’ survival and the impact of time to surgery on gastric cancer outcomes is uncertain.

The results of the present meta-analysis show that delaying surgery for less than 8 weeks may not decrease OS in gastric cancer. We did not find the longest waiting time to perform a surgery for gastric cancer patients. A subgroup analysis also did not show a significant association between delayed surgery and worsening survival. To our knowledge, there is no specific guidelines existing for an appropriate time interval for oncological surgery for gastric cancer patients (21). Studies from China, North America, and Europe have shown that patients with cancer have a higher risk of severe clinical events and mortality, which was compared with no-cancer patients (22). During SARS-CoV-2 outbreaks, consideration should be given for promoting non-operative treatment to delay the need for surgery (23). However, there is not enough evidence to definitively link chemotherapy, radiotherapy, and immunotherapy with increased risk of death (24). During the COVID-19 pandemic, anticancer treatment would be deferred for 14 days in patients who test positive for SARS-CoV-2 (25).

Gastric cancer is a common malignancy around the world and is also extremely lethal (26). For this, it is of equal importance in the time to surgery and diagnosis of gastric cancer. Currently, the COVID-19 pandemic has been disrupting the screening endoscopy including gastroscopy, colonoscopy, and even oncology surgery (27). We conducted the meta-analysis with the aim to evaluate the impact of delay of the gastrectomy on gastric cancer patients’ survival outcomes. Compared with delaying less than 4 weeks, delaying for a longer time (more than 4 weeks and less than 8 weeks) cannot decrease the overall survival and disease-free survival. A recent study by Wang et al. (28) suggested that immunotherapy and chemotherapy are not associated with increased risk of mortality in patients with cancer during the COVID-19 pandemic, which is similar to our study.

There are several limitations in our meta-analysis. Considering that gastric cancer is a progressive disease, increasing the delaying time to surgery should result in worse clinical outcomes including overall survival. Therefore, our meta-analysis cannot answer the question that an accurate delay time to surgery affects the patients’ survival data. All of the included studies were retrospective studies, which showed a selection bias in the included patients. Even though the overall assessment of quality indicates the inclusion of high-quality studies, there were numerous studies which did not control for stage and/or did not have an appropriate length of follow-up.

## Conclusion

The results of the meta-analysis suggest that a delay in surgery of less than 8 weeks is not associated with worse overall survival for patients with gastric cancer. To help better guide surgical decisions during the COVID-19 pandemic, further studies should be designed as multicenter, prospective, and randomized studies. Our conclusions also need a high-quality random-controlled trial to be evaluated.

## Data Availability Statement

The original contributions presented in the study are included in the article/supplementary material. Further inquiries can be directed to the corresponding authors.

## Author Contributions

Study concept and design: MD, LG, JM. Acquisition of data: JM, CZ, WL. Analysis and interpretation of data: all authors. Drafting of manuscript: JM and CZ. Critical revision of the manuscript for intellectual content: all authors. Final approval of the version to be published: All authors. Accountable for all aspects of the work: JM. All authors contributed to the article and approved the submitted version.

## Funding

This research was supported by the National Natural Science Foundation of China (No. 82160588).

## Conflict of Interest

The authors declare that the research was conducted in the absence of any commercial or financial relationships that could be construed as a potential conflict of interest.

## Publisher’s Note

All claims expressed in this article are solely those of the authors and do not necessarily represent those of their affiliated organizations, or those of the publisher, the editors and the reviewers. Any product that may be evaluated in this article, or claim that may be made by its manufacturer, is not guaranteed or endorsed by the publisher.
